# Anode Biofilm Transcriptomics Reveals Outer Surface Components Essential for High Density Current Production in *Geobacter sulfurreducens* Fuel Cells

**DOI:** 10.1371/journal.pone.0005628

**Published:** 2009-05-20

**Authors:** Kelly P. Nevin, Byoung-Chan Kim, Richard H. Glaven, Jessica P. Johnson, Trevor L. Woodard, Barbara A. Methé, Raymond J. DiDonato, Sean F. Covalla, Ashley E. Franks, Anna Liu, Derek R. Lovley

**Affiliations:** 1 Department of Microbiology, University of Massachusetts, Amherst, Massachusetts, United States of America; 2 Department of Mathematics, University of Massachusetts, Amherst, Massachusetts, United States of America; 3 J. Craig Venter Institute, Rockville, Maryland, United States of America; University of Massachusetts Medical School, United States of America

## Abstract

The mechanisms by which *Geobacter sulfurreducens* transfers electrons through relatively thick (>50 µm) biofilms to electrodes acting as a sole electron acceptor were investigated. Biofilms of *Geobacter sulfurreducens* were grown either in flow-through systems with graphite anodes as the electron acceptor or on the same graphite surface, but with fumarate as the sole electron acceptor. Fumarate-grown biofilms were not immediately capable of significant current production, suggesting substantial physiological differences from current-producing biofilms. Microarray analysis revealed 13 genes in current-harvesting biofilms that had significantly higher transcript levels. The greatest increases were for *pilA*, the gene immediately downstream of *pilA*, and the genes for two outer *c*-type membrane cytochromes, OmcB and OmcZ. Down-regulated genes included the genes for the outer-membrane *c*-type cytochromes, OmcS and OmcT. Results of quantitative RT-PCR of gene transcript levels during biofilm growth were consistent with microarray results. OmcZ and the outer-surface *c*-type cytochrome, OmcE, were more abundant and OmcS was less abundant in current-harvesting cells. Strains in which *pilA*, the gene immediately downstream from *pilA*, *omcB*, *omcS*, *omcE*, or *omcZ* was deleted demonstrated that only deletion of *pilA* or *omcZ* severely inhibited current production and biofilm formation in current-harvesting mode. In contrast, these gene deletions had no impact on biofilm formation on graphite surfaces when fumarate served as the electron acceptor. These results suggest that biofilms grown harvesting current are specifically poised for electron transfer to electrodes and that, in addition to pili, OmcZ is a key component in electron transfer through differentiated *G. sulfurreducens* biofilms to electrodes.

## Introduction

Previous attempts to improve the power output of microbial fuel cells have primarily focused on overcoming electrochemical limitations, but a better understanding of how microorganisms transfer electrons to electrodes could lead to the design of microorganisms with improved electron-transfer capabilities [1]–[4].

A wide diversity of microorganisms are capable of electron transfer to electrodes [5], [6]. We have focused our studies on *Geobacter sulfurreducens* because this species, or closely related organisms, are frequently the most abundant microorganisms colonizing anodes harvesting current from aquatic sediments [7]–[9] and in laboratory fuel cells designed for high current densities under highly anoxic conditions [10]–[13].

Experimental manipulation [14], electrochemical analysis [15], [16] and modeling studies [17] have all suggested that *G. sulfurreducens* transfers electrons to anodes via bound mediator(s) rather than soluble electron shuttles. The nature of the bound mediator(s) is a matter of active investigation. In early studies, in which current levels were low (0.08–0.39 A/m^2^), anode biofilms were thin with most cells closely associated with the anode surface [14]. Gene expression analysis and genetic studies suggested that the outer-surface cytochromes OmcS and OmcE might be the involved in electron transfer to the anode surface in such systems [18]. Electrically conductive pili, commonly referred to as microbial nanowires [19], were not required for this low-density current production [18].

In subsequent studies, higher current densities (4.56 A/m^2^ **)** were achieved, primarily by supplying additional electron donor, most preferably on a continuous basis in anode chambers with a continual input of fresh medium [20], [21]. Under these conditions biofilms of ca. 50 µm in thickness were produced on flat graphite surfaces [20], [21]. Despite the fact that most of the cells were no longer in direct contact with the anode, there was a direct correlation between biomass on the anode and current during the initial phase of biofilm formation which suggested that microorganisms at substantial distance from the anode could significantly contribute to current production [20]. Deleting the gene for PilA, the structural pilin protein, eliminated the capacity for high-density current production, suggesting that pili are required for long-range electron transfer through the biofilm [20].

However, *Geobacter sulfurreducens* contains many additional potentially redox-active proteins that could contribute to high-density current production. The purpose of the study reported here was to identify potential candidates via whole-genome gene expression analysis and evaluate these candidates with genetic approaches.

## Methods

### Culture

*Geobacter sulfurreducens* strain PCA (ATCC 51573, DSMZ 12127) was obtained from our laboratory culture collection. The inoculum for the growth on electrodes was grown as previously described in NBAF medium (0.04 g/L CaCl_2_*2H_2_O, 0.1 g/L MgSO_4_*7H_2_O, 1.8 g/L NaHCO_3_, Na_2_CO_3_*H_2_O, 0.42 g/L KH_2_PO_4_, 0.22 g/L K_2_HPO_4_, 0.2 g/L NH_4_Cl, 0.38 g/L KCl, 0.36 g/L NaCl, vitamins and minerals) [22] with acetate (10 mM) as the electron donor and fumarate (40 mM) as the electron acceptor with the resazurin omitted and with 1.3 mM Fe(II) chloride or 1 mM cysteine added as a reductant.

### Mutant Construction and complementation

The *pilA* deletion mutant and *pilA* complement [20], as well as the *omcB* [23], *omcS* [24], and *omcE* [24] deletion mutants were constructed as previously described. The gene for OmcZ (GSU2076) was deleted with the previously described [25] single-step gene replacement method. A linear 2.1-kb DNA fragment, containing the kanamycin resistance marker (Kan^r^) flanked by ca. 0.5 kb of sequence upstream and downstream of *omcZ* was generated by recombinant PCR [25], [26]. The sequence upstream of *omcZ* was amplified with primers 2076-1 and 2076-2 (Table 1). The sequence downstream of *omcZ* was amplified with primers 2076-5 and 2076-6. The kanamycin resistance cassette was amplified from plasmid pBBR1MCS-2 [27] with primers 2076-3 and 2076-4 recombinant PCR with the three primary PCR products, the final 2.1-kb fragment was amplified with distal primers 2076-1 and 2076-6. To delete GSU 1497, the primers1497rg1 and 1497rg2R1 (Table 1) were used to amplify a 500 bp fragment upstream from ORF GSU1497 and 1497rg3H3 and 1497rg4C 500 bp fragment downstream from ORF GSU1497. The kanamycin resistance cassette was amplified from plasmid pBBR1MCS-2 with primers rgKANR15′ and rgKANRevH3. Fragments were combined and double digested with EcoRI/HindIII (NEB) and ligated (Epicenter). The ligation reaction was purified with the QIAquick PCR Purification Kit (Qiagen) and amplified with primers 1497rg1 and 1497rg4C. The PCR conditions were similar to those described previously [28]. Electroporation, mutant isolation, and genotype confirmation were performed as previously described [22], [25]. Mutant strains DLBK17 (*omcZ*::*kan*) and DLRG9 (*GSU1497::kan*) were generated. The genotypes of these strains were confirmed by DNA sequencing of genomic DNA of each mutant strain.

**Table 1:** Primers used for mutant construction, complementation and RT-PCR analyses.

Gene	Purpose	Primer Name	Primer Sequence (5′ - 3′)
*omcZ*	mutant construction	2076-1	ATGTGATGCGATATCCCGGC
		2076-2	CGCTGACGTGACACTCGAGAC
		2076-3	GTCTCGAGTGTCACGTCAGCGAGTGCCACCTGGGATGAATG
		2076-4	GGTGATGCGGAGCTCGTAGATGGCAGGTTGGGCGTCGC
		2076-5	CTACGAGCTCCGCATCACC
		2076-6	CACCCAGAGGAGGCAGCAGG
GSU1497	mutant construction	1497rg1	CGAACTTTTGAAGCTTACGTTCC
		1497rg2R1	GATAGCGGAATTCCATGGTTTCCTCCAGTATG
		1497rg3H3	GTATGCAAGCTTTAGTCATATCGGATAACTGATTG
		1497rg4C	GAAACCCACACCACAGATTCCGTATTGAGCG
		rgKANR15′	GCATGAGAATTCCTGACGGAACAGCGGGAAGTCCAGC
		rgKANRevH3	GCTATGAAGCTTTCATAGAAGGCGGCGGTGGAATCGAA-
		Rg2076R1	GCATATGAATTCAATTCAAGAAAGGAGCAGAAAGGAATG
		Rg2076H3	GCATCAAAGCTTTACCGTTTGACTTTCTTCGGAGC
GSU2075 & *omcZ*	operon testing	2076RT-F	CCCAGGCAAACGTCATTTCC
		2075RT-R	CAATGGACGAAAGCACGAAG
*pilA*	q-RT	RT_ORF02545_F	CCAACACAAGCAGCAAAAAG
		RT_ORF02545_R	GCAGCGAGAATACCGATGAT
*omcB*	q-RT	RT_ORF04536_F	GACACGGTCAACCAGAACAA
		RT_ORF04536_R	GGTCCCAGTTTACGACAGGA
*omcE*	q-RT	RT_ORF01030_F	CTCGTCCAGCAGCATGAATA
		RT_ORF01030_R	GGGGTGATCATTGCTCAGAT
*omcS*	q-RT	RT_ORF04142_F	CAACCTGGCATACGAGTTCA
		RT_ORF04142_R	CCATAGTAGGCAGCGGTCAT
*omcZ*	q-RT	RT_ORF03438_F	CACGAGCCTGACACTCACTC
		RT_ORF03438_R	AAGGTTGCTGACCTTGTTGG
GSU1497	q-RT	RT_ORF02546_F	ATGGGTGGCAAGGACTTTA
		RT_ORF02546_R	AACACCCGGTTACCAGAAGA

To complement the *omcZ* mutation, primers Rg2076R1 and Rg2076H3 were used to amplify the gene coding for OmcZ and its ribosome binding site from *G. sulfurreducens* chromosomal DNA. (Epicenter Biotechnologies MasterPure DNA purification kit. Madison, WI53713). The resulting PCR product was purified with the QIAquick PCR Purification Kit (Qiagen) and digested with EcoR1 and HindIII (New England Biolabs Beverly, MA) and ligated in to previously EcoR1/HindIII digested expression vector pRG5 [29].

### Growth on Electrodes

Graphite electrodes (65 cm^2^ solid graphite blocks, 1 in. by 0.5 in. by 3 in., grade G10, , Graphite Engineering and Sales, Greenville, MI), poised with a potentiostat (+300 mV versus Ag/AgCl) in ‘H-type cells’ were provided as an electron acceptor as previously described [7], [14]. The cells were grown in freshwater medium (2.5 g/L NaHCO_3_, 0.25 g/L NH_4_Cl, 0.06 g/L, NaHPO_4_*H_2_O, 0.1 g/L KCl, vitamins and minerals) [30] containing 10 mM acetate and 40 mM fumarate. When the culture reached an A_600_ of 0.2, the anode chamber was swapped to freshwater medium containing 10 mM acetate and no fumarate. Once the current reached approximately 1 mA the system was switched to a continuous flow through mode, in which medium was flowed through the chamber at a dilution rate of 0.15/hr. Current measurements were collected directly from potentiostat outputs every second with a Power Lab 4SP connected to a Macintosh computer, and data was logged with Chart 5.0 software (ADI instruments, Mountain View, CA). Fumarate control fuel cells were grown as described above but with the working, counter and reference electrodes disconnected and with 40 mM fumarate present in the medium.

### Microarray culture conditions, RNA extraction and treatment

Current-harvesting biofilms were grown until the current reached 10 mA and fumarate controls were grown until a biofilm was visible (∼4 days). The biofilms were harvested by scraping and RNA extracted as previously described [18]. Extracted nucleic acids were treated for DNA contamination using the DNA-free Kit (Ambion) according to the manufacturer's suggested protocol and tested for genomic DNA contamination by polymerase chain reaction for 40 cycles (95°C 3 min; 95°C 15 sec, 58°C 30 sec, 72°C 90 sec; with a final extension of 72°C 10 min) using primers specific to *G. sulfurreducens*. Ten percent of the PCR reaction was analyzed on a 0.8% agarose/TAE gel stained with ethidium bromide. If amplification products indicating contaminating genomic DNA were detected, then the samples received additional DNase treatment.

Ten micrograms of total RNA was chemically labeled with Cy3 (fumarate-grown cells) or Cy5 (electrode-grown cells dyed using the MicroMax ASAP® RNA Labeling Kit (Perkin Elmer, Wellesley, MA) according to manufacturer's instructions. Labeled RNA was then washed four times with 200 µl RNase-free H_2_O in MicroCon spin columns (Millipore, Billerica, MA) and subsequently subjected to fragmentation with the Fragmentation Reagent (Ambion) in a 20 µl volume at 70°C for 30 minutes. Control and experimental RNA were then combined, ethanol precipitated, washed three times with 70% ethanol, and resuspended in 20 µl of nuclease-free H_2_O.

### Microarray Analysis

As previously described [31] Customarray™ 12K arrays (Combimatrix, Mukilteo, WA) were hybridized per the manufacturers instructions (Combimatrix, Mukilteo, WA). The arrays were scanned using a Genepix 4000B scanner (Molecular Devices Inc., Sunnyvale, CA), and analyzed using GenePix and Acuity 4.0 software. To minimize background effects, square outlines were utilized during spot finding to reduce the variance in the contribution of the local background signal. As each spot or outlined spot contains the same area, the total intensity from each spot for each channel is calculated as opposed to the more traditional mean intensity. Data obtained are then exported into the R package for further statistical analysis.

The raw total intensities were treated as previously described to generate the log_2_ ratios (M) where [M = log_2_ (Ex/Ct)] [31]. During the data preprocessing, Ratio versus Intensity or MA plots before and after normalization, and side-by-side box plots for all arrays were used to assess array quality[32]. LIMMA mixed model analysis (R-package LIMMA[33]) was applied to the normalized logged ratios to identify differentially expressed genes as previously described [31]. The GEO number for the array data is GSE8012 (http://www.ncbi.nlm.nih.gov/geo/query/acc.cgi?token=xveldmmyoswqane&acc=GSE8012). Genes with fold change of greater than 1.5 were included in analyses.

### RT-PCR and qRT-PCR analysis

The DuraScript enhanced avian RT single-strand synthesis kit (Sigma-Aldrich Co. St Louis, MO) was used to generate cDNA with random primers according to the suggested protocols (Sigma-Aldrich Co. St Louis, MO). The cDNA generated by RT-PCR was used as template for PCR analysis to determine whether GSU2076 (*omcZ*) and GSU2075 are part of the same operon. The primers, 2076RT-F and 2075RT-R (Table 1) amplified a segment of the cDNA extended from the 3′ end of GSU2076 (*omcZ*) into the 5′ end of GSU2075.

Quantitative real time PCR (qRT-PCR) was used to monitor the expression of six experimental genes as follows. Reactions were performed in triplicate for each gene tested from one biological sample representing each level of current and compared to a reference sample of cells growing in suspension using acetate as the electron donor and a chelated source of iron (ferric citrate) as the electron acceptor. A reverse transcription reaction was performed to synthesize single-stranded cDNA from approximately one microgram of total RNA from each sample in a 100 µl reaction volume using the Taqman® Reverse Transcription Reagents (Applied Biosystems, Foster City, CA). The resulting cDNA was subsequently used as template for real-time PCR using the SYBR® Green PCR Master Mix (Applied Biosystems) and primers suitable for qRT-PCR amplification that were designed using Primer3 software [34]. Forward and reverse primers were added to the reaction at a final concentration of 200 nM along with one microliter of the cDNA reaction. The incorporation of SYBR Green dye into the PCR products was detected in real time on the ABI Prism 7900HT Sequence Detection System. The ROX passive reference dye was used to normalize for non-PCR related fluorescence signal variation (e.g., well and pipetting variability). The resulting incorporation of the SYBR Green was used to determination the threshold cycle (Ct), which identifies the PCR cycle at which exponential production of the PCR amplicon begins. Relative expression levels were calculated by the 2^−ΔΔCT^ method [35]. The PCR primers are in Table 1.

### Detection of loosely bound outer membrane *c*-type cytochromes

Fuel cells were grown to 10 mA of current produced and fumarate biofilm control cells were harvested as described above. Wild type *G. sulfurreducens* and OmcZ-deficient mutant cells grown in NBAF media [22] were harvested when they reached late log phase. The loosely bound outer membrane-enriched protein fractions (LBOP) of the cells were isolated as previously described [24] and were separated by Tris-Tricine denaturing polyacrylamide gel electrophoresis (PAGE) followed by staining with *N,N,N′,N′*-tetramethylbenzidine as previously described [36], [37]. The differently expressed cytochrome bands from Tris-Tricine denaturing polyacrylamide gel were excised and sent to the Laboratory for Proteomic Mass Spectrometry in the University of Massachusetts Medical School for LCMS/MS analysis in order to identify cytochromes.

### Other Analyses

Operon predictions were described in an earlier report [38]. The version of G. *sulfurreducens* operon annotation of May 2007 was used. Protein was measured with the bicinchoninic acid method (Sigma, St. Louis, MO) as previously described [21]. Acetate and other organic acids were determined via HPLC with a fast-acid column (Bio-Rad, Hercules, CA) with an eluent of 8 mm H_2_SO_4_ and UV detection at 210 nm. Confocal microscopy was performed on biofilm samples as previously described using LIVE/DEAD® BacLight™ Stain [20], [21].

## Results

### Differences in Electron Transfer Properties of Biofilms Producing Current or Reducing Fumarate

**Figure 1:**
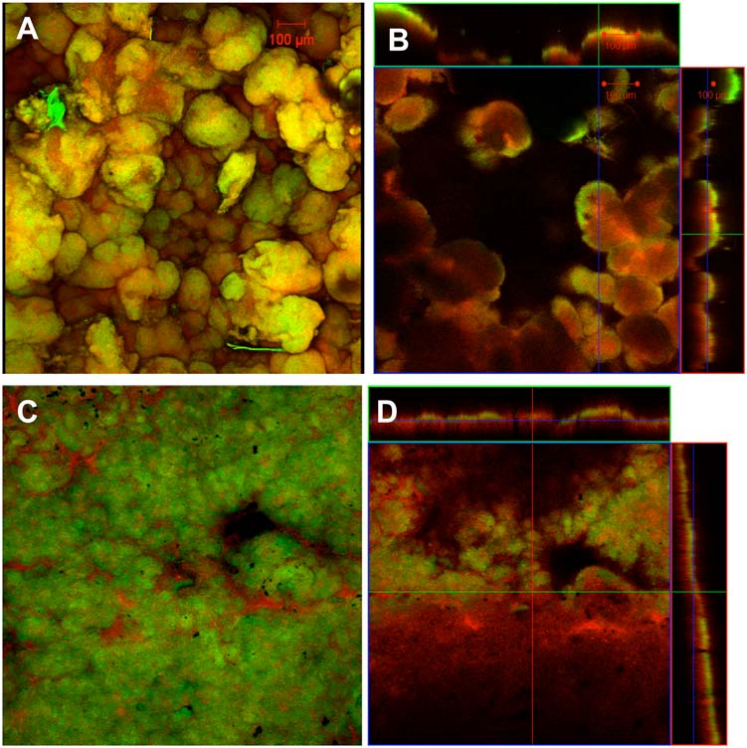
Confocal scanning laser microscopy images of *Geobacter sulfurreducens* grown with different electron acceptors. Confocal scanning laser microscopy images of current harvesting and fumarate control biofilms of wild type *G. sulfurreducens.* Metabolically active (green) and inactive (red) cells where differentiated with a LIVE/DEAD kit based on the permeability of the cell membrane. A. 3-D projection, top view, fumarate control biofilm; B. slices through biofilm parallel to electrode large panel and perpendicular to electrode top and side panel, fumarate control biofilm; C. 3-D projection, top view, current harvesting biofilm; D. slices through biofilm parallel to electrode large panel and perpendicular to electrode top and side panel, current harvesting biofilm.

Previous studies demonstrated that *G. sulfurreducens* produced a biofilm on graphite surfaces that served as an electron acceptor in microbial fuel cells [20]. In order to determine if a biofilm would grow on the same graphite surface with an alternative electron acceptor, cells were grown in the presence of the same graphite surface, but electrically disconnected from the cathode, preventing electron transfer to the graphite. Fumarate was provided as an alternative electron acceptor. The fumarate-respiring cells formed a thick, highly structured biofilm on the graphite (Fig. 1a & 1b). Fumarate-grown biofilms had more biomass, appeared more “fluffy”, and were less tightly associated with the graphite surface than the biofilms that were grown in current-harvesting mode with the graphite serving as the electron acceptor (Fig. 1c & 1d).

**Figure 2:**
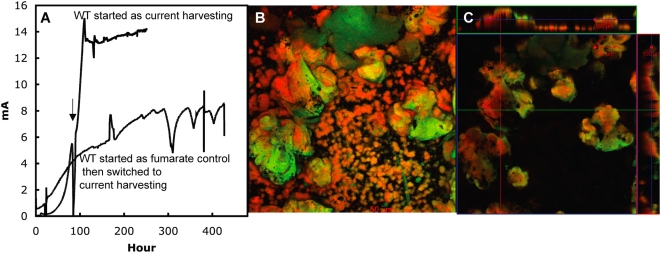
Current production of *Geobacter sulfurreducens*. A. Current production time courses of wild type *G. sulfurreducens* grown entirely as current harvesting (arrow indicates the switch from original feed of 10 mM acetate to continual feed) and current production time course of fully grown fumarate control biofilms switched to current harvesting of wild type *G. sulfurreducens*. These data are representative time courses for multiple replicates of each treatment. B–C. Confocal scanning laser microscopy images of fumarate control swapped to current harvesting biofilms of *G. sulfurreducens* . Metabolically active (green) and inactive (red) cells where differentiated with a LIVE/DEAD kit based on the permeability of the cell membrane. B. 3-D projection, top view; C. slices through biofilm parallel to electrode large panel and perpendicular to electrode top and side panel.

In order to determine whether the cells of the fumarate-grown biofilm were poised for current production, the medium of a fumarate-grown biofilm that had reached a thickness of ca. 100 µm was replaced with medium containing no fumarate and the graphite electrode was connected to the cathode. Despite the abundance of cells in the biofilm, the current was only 0.6 mA (Fig. 2a). The current increased slowly, eventually reaching 8 mA, but only after 270 hours of operation. In contrast, starting with an uncolonized electrode and introducing only a small inoculum of fumarate-grown cells produced ca. 14 mA within 110 hours of operation (Fig. 2a).

Examination of the biofilm initially grown on fumarate and then switched to the electrode as the electron acceptor revealed large pillars, typical of those in fumarate-grown biofilms, surrounded by smaller pillars similar to those seen in other current-harvesting biofilms (Fig. 2b). In cross-sectional views the large pillars that presumably grew in the initial growth phase with fumarate as the electron acceptor contained a mix of red and green cells in the center with an outer coating of green cells. The smaller pillar also contained a mixture of red and green cells. Green cells are considered to be metabolically active, whereas those staining red may have compromised membranes, possibly viable, but non-growing [39], [40].

### Differences in Gene Transcript Levels In Current-Producing and Fumarate-Reducing Biofilms

In order to identify differently expressed genes associated with the apparent physiological differences in biofilms growing with the electrode or fumarate as the electron acceptor, transcript levels in current-harvesting biofilms growing on a graphite electrode were compared with transcript levels in biofilms growing on the same graphite surface, but with fumarate as the electron acceptor. Only 13 genes had significantly higher transcript levels in current-harvesting mode (Table 2) and 10 genes had significantly lower transcript levels (Table 3).

**Table 2:** Gene transcripts whose expression (arithmetic) was significantly increased when *G. sulfurreducens* was grown within a current-harvesting 10 mA potentiostat biofilm versus a fumarate control biofilm.

Locus ID	Common Name	Role Category/Main Role	Subrole	fold change
GSU1497	hypothetical protein			8.05
GSU1496	*pilA*, pilin domain protein	Unknown function	General	6.03
GSU2737	*omcB*, polyheme membrane-associated cytochrome c	Energy metabolism	Anaerobic	6.00
GSU2076	*omcZ*, cytochrome c family protein	Energy metabolism	Electron transport	5.70
GSU3401	branched-chain amino acid ABC transporter, periplasmic amino acid-binding protein, putative	Transport and binding proteins	Amino acids, peptides and amines	5.03
GSU2733	hypothetical protein, in operon with *omcC*			4.02
GSU2732	cytochrome c family protein, in operon with *omcC*	Energy metabolism	Electron transport	2.78
GSU3403	hypothetical protein			2.76
GSU2739	hypothetical protein, in operon with *omcB*			2.47
GSU2075	subtilisin	Protein fate	Degradation of proteins, peptides, and glycopeptides	2.08
GSU3406	amino acid ABC transporter, periplasmic amino acid-binding protein	Transport and binding proteins	Amino acids, peptides and amines	1.90
GSU0975	phage tail sheath protein, putative	Other categories	Prophage functions	1.82
GSU3402	hypothetical protein			1.63

Limma Analysis with a maximum p value of 0.001 was used for statistical analysis.

**Table 3:** Gene transcripts whose expression (arithmetic) was significantly decreased when *G. sulfurreducens* was grown within a current-harvesting 10 mA potentiostat biofilm versus a fumarate control biofilm.

Locus ID	Common Name	Role Category/Main Role	Subrole	fold change
GSU2504	*omcS*, cytochrome c family protein	Energy metabolism	Electron transport	−4.53
GSU2780	hypothetical protein			−3.71
GSU2780b	hypothetical protein			−3.16
GSU1333	hypothetical protein			−2.90
GSU2751	*dcuB*, C4-dicarboxylate transporter, anaerobic	Transport and binding proteins	Carbohydrates, organic alcohols, and acids	−2.66
GSU1340	ABC transporter, permease protein	Transport and binding proteins	Unknown substrate	−2.17
GSU1330	metal ion efflux outer membrane protein family protein, putative	Cellular processes	Detoxification	−2.00
GSU2503	*omcT*, cytochrome c family protein	Energy metabolism	Electron transport	−1.96
GSU2750	conserved domain protein	Hypothetical protein	Domain	−1.68
GSU1330b	metal ion efflux outer membrane protein family protein, putative	Cellular processes	Detoxification	−1.63

Limma Analysis with a maximum p value of 0.001 was used for statistical analysis. Genes included has log2 ratios of greater than 1.

The greatest increases in transcript levels in cells growing with the electrode as the electron acceptor were in the gene for the structural subunit of pilin, *pilA* and for GSU1497, which is adjacent to *pilA* and is annotated as a conserved hypothetical protein (Table 2). Next in greatest relative increase in transcript level abundance was the gene for OmcB, an outer-membrane *c*-type cytochrome that is known to be important in Fe(III) reduction [23], [41], [42]. GSU2739, which is in an operon with *omcB* and encodes a hypothetical protein [43], also had higher transcript levels in the current-harvesting biofilm as did a nearly identical gene, GSU2732, as well as GSU2733, in an operon of the *omcB* homolog, *omcC* [43].

**Figure 3:**
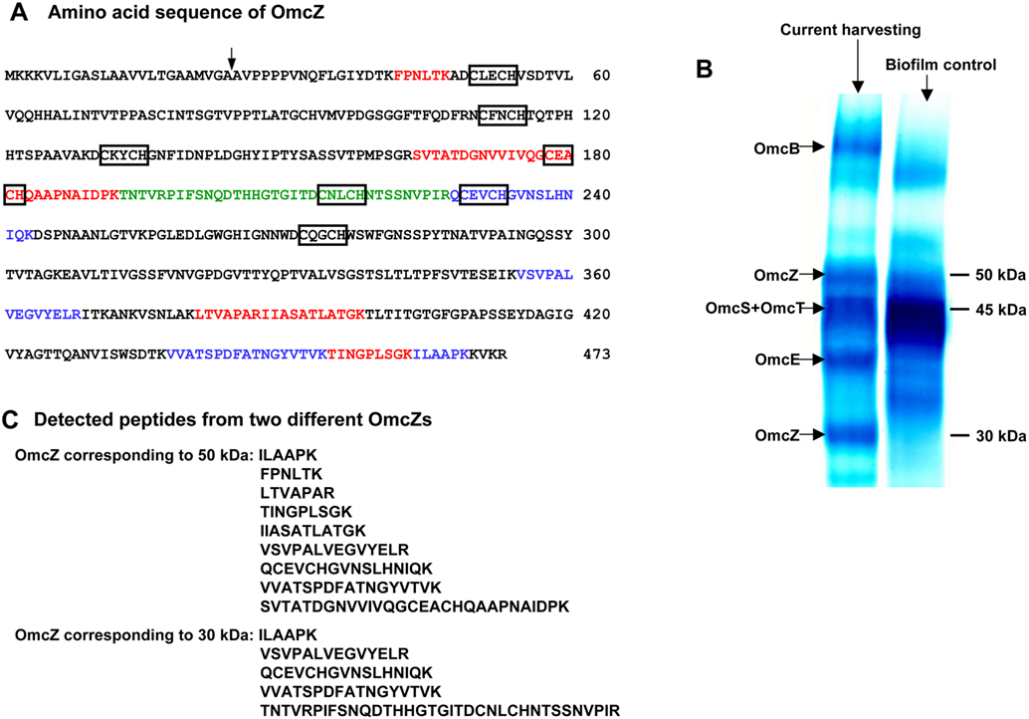
OmcZ sequence and identification. A. Amino acid sequence of OmcZ. The predicted cleavage site for mature OmcZ indicated by arrow. Confirmed heme-binding domains (CXXCH) are enclosed in boxes. OmcZ has 473 amino acid residues and 7 heme binding domains. B. Cytochrome content of loosely bound outer membrane protein-enriched fractions from fumarate and current-harvesting biofilms. Proteins (10 µg/lane) were separated by 12% Tris-Tricine denaturing polyacryamide gel electrophoresis and stained for heme. C. Peptides detected from two different sizes of OmcZs, fragments detected in the 50 KDa OmcZ are indicated in panel A in red; fragments detected in the 30 KDa OmcZ are indicated in panel A in green and fragments detected in both size OmcZ are indicated in blue.

GSU2076 also had significantly higher transcript levels in current-harvesting mode (Table 2). This gene is predicted to encode a *c*-type cytochrome, with 7 heme groups and a molecular weight of 49kDa (Fig. 3). PSORT (http://psort.nibb.ac.jp./form.html) predicted that this cytochrome should be localized in the outer membrane. Therefore this protein was designated outer membrane cytochrome Z (OmcZ). GSU2075, which is predicted to encode a subtilisin enzyme (a proteolytic enzyme, serine endopeptidase) and which is in an operon with *omcZ* [38] also had more abundant transcripts in the current-producing biofilm.

Higher transcript levels were also observed for several genes that are predicted to encode functions other than electron transfer. These included three genes predicted [38] to be in an operon: GSU3401, which is predicted to be involved in amino acid transport; GSU3402, encoding a hypothetical protein; and GSU3403, also encoding a hypothetical protein. GSU3406, which is also predicted to be involved in amino acid transport, had slightly higher transcript levels in current-harvesting cells.

The greatest decrease in transcript level was for the outer membrane *c*-type cytochrome gene *omcS* (Table 3). Transcript levels for another outer-membrane cytochrome gene, *omcT*, were also lower. The *omcT* gene is co-transcribed with *omcS*, but monocistronic transcripts of *omcS* are also generated [18], [24]. Transcript levels for *dcuB*, which encodes the fumarate transporter [44], were lower in current-producing cells as was the gene for the hypothetical protein in the same operon, reflecting the fact that fumarate was not being used as an electron acceptor. Transcript levels were also somewhat lower for several genes encoding hypothetical proteins: a putative metal ion efflux outer membrane protein; a permease component of an ABC transporter; and a putative oxidoreductase of unknown function (Table 3).

The microarray analysis only provided an indication of relative transcript levels in the mature biofilms continuously producing 10 mA current. In order to obtain more information on changes in transcript levels during growth on the anode, transcript levels for several genes encoding outer-surface proteins were monitored over time at different levels of current output and biofilm thickness. The analysis included those genes that the microarray analysis indicated were significantly up-regulated in current-producing cells as well as the gene for OmcE. Although transcripts for *omcE* were not significantly different in current-producing and fumarate-grown biofilms at the high stringency value (p value .001), at a lower stringency level (p value .05) there did appear to be slightly higher transcript levels for *omcE* in the current-harvesting cells. Furthermore, higher transcripts for *omcE* were previously noted at lower current densities [18]. Planktonic cells grown with Fe(III) citrate as the electron acceptor served as the comparator because: 1) it was too technically difficult to match biofilm thickness in biofilms grown with fumarate and the electrode serving as the electron acceptor; and 2) levels of *omcS* expression are low during growth on Fe(III) citrate, providing the opportunity to more readily discern differences in *omcS* transcript levels in the electrode biofilms.

**Figure 4:**
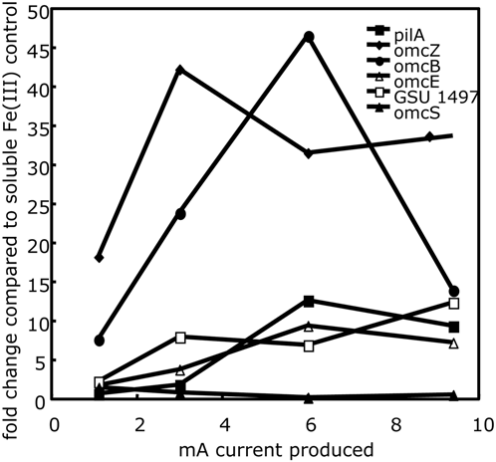
RT-PCR of genes up-regulated in microarray analyses. Fold change of *pilA*, *omcB*, *omcE*, *omcS*, *omcZ* and GSU1497 at different amounts of current produced compared to a soluble Fe(III) control as determined by quantitative RT-PCR.

Transcripts for most of the genes evaluated increased in abundance with increased current/biofilm thickness up to 3–6 mA, then remained relatively steady or, in the case of *omcB* transcripts, declined (Fig. 4). The exception was transcripts of *omcS*, which steadily declined with increased current/biofilm thickness.

### Analysis of Outer-Surface Cytochrome Abundance

In order to determine whether the changes in transcript levels for genes encoding predicted outer-surface *c*-type cytochromes influenced the abundance of *c*-type cytochromes on the outer surface of the cells, loosely bound outer membrane proteins from the current-harvesting and fumarate control biofilms were separated with Tris-Tricine SDS-PAGE and stained for heme (Fig. 3B). The cytochrome patterns were distinctly different. The fumarate control contained an intensely stained band corresponding to the position of the previously described [24] outer-surface *c*-type cytochrome, OmcS, and its homologue, OmcT. This band was less intense in the current-harvesting biofilm cells. LCMS/MS analysis of this band in both types of cell samples revealed that the most abundant peptides were from OmcS, but two peptides specific for OmcT were also detected. These were the only cytochrome peptides detected in this band.

There were four bands that were much more prominent in the sample of the current-harvesting cells. Two of these bands correspond to the intensively studied outer-membrane proteins OmcB [23], [28] and OmcE [24]. Higher abundance of these cytochromes in the current-harvesting cells was consistent with the higher levels of their gene transcripts. LCMS/MS analysis of the two additional prominent bands at ca. 50 and 30 kDa in the current-harvesting sample yielded OmcZ, as the only cytochrome peptide (Fig. 3C). Subsequent studies with purified OmcZ have demonstrated that the 50 kDa band contains the full length protein whereas the smaller protein at 30 kDa results from cleavage of a portion of the carboxyl terminal portion of the protein (K. Inoue, personal communication).

### Impact of Gene Deletions on Current Production and Biofilm Formation

**Figure 5:**
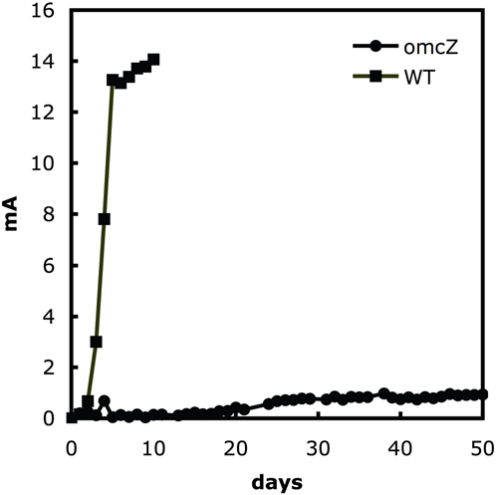
Current production of *Geobacter sulfurreducens* wild type and *omcZ* mutant. Current production time course of wild type and *omcZ*-deletion mutant. The data is a representative time course for multiple replicates of each treatment.

The role of outer-surface proteins whose genes were differentially expressed in the current-producing biofilm versus the biofilm reducing fumarate were further evaluated by deleting each gene and determining its impact on current production. Deletion of *omcS*, *omcB*, or *omcE* had no impact on maximum current production (Table 4). The strain in which GSU1497 was deleted had maximal current production that was 2/3rds that of wild type (Table 4), but took 5 times longer to reach its maximum than wild-type cells. Deletion of *pilA* or *omcZ* significantly reduced power production. Neither the *omcZ*-deficient mutant (Fig. 5) nor the *pilA*-deficient mutant [20] adapted to produce higher power, even after extended incubations of more than 50 days. When these mutations were complemented by expressing *pilA* or *omcZ* on a plasmid, wild type levels of current production were restored (Table 4). Previous studies demonstrated that deletion of *pilA* did not impact on growth with fumarate or Fe(III) citrate as the electron acceptor, but that growth on Fe(III) oxide was severely inhibited [19]. Deletion of *omcZ* had no impact on growth with any of these electron acceptors (data not shown).

**Table 4:** Maximal current production of fuel cells inoculated with wild-type cells of *G. sulfurreducens* or strains with the designated genes deleted or the deleted genes complemented via expression of the designated gene on a plasmid.

Genetic Manipulation	Maximum Current Production (mA)
Wild Type	14.42±0.62
*pilA* deletion	1.20±0.44
*pilA* complement	14.12±0.14
*omcZ* deletion	1.3±0.36
*omcZ* complement	13.44±0.37
*omcB* deletion	12.41±0.63
*omcE* deletion	12.75±0.41
*omcS* deletion	12.67±0.57
GSU1497 deletion	8.01±0.42

Values are the average±the standard deviation of triplicates.

**Figure 6:**
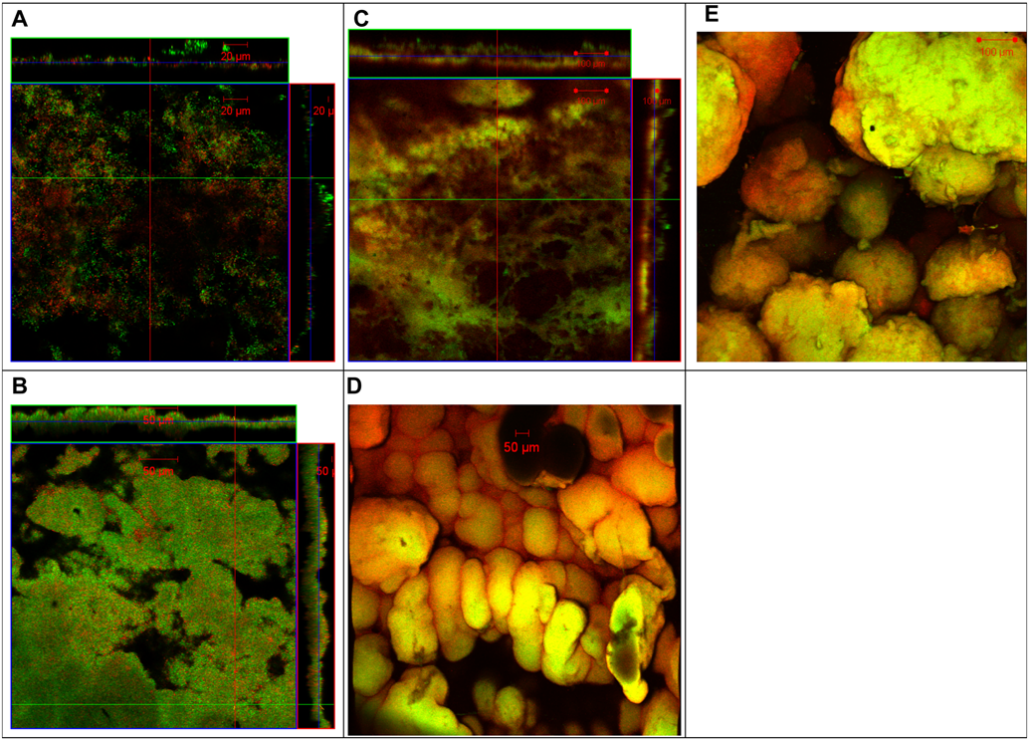
Confocal scanning laser microscopy images of *Geobacter sulfurreducens* mutants. Confocal scanning laser microscopy images of current harvesting mutant biofilms and complemented strains . Cell stained green indicate metabolically active, cells strain red indicate metabolically inactive. A–C. slices through biofilm parallel to electrode large panel and perpendicular to electrode top and side panel; A. *omcZ* mutant, B. *omcZ* complement, C. *pilA* complement; D–E. 3-D projection, top view, fumarate control biofilms, D. *omcZ* mutant, E. *pilA* mutant.

The biofilm of the *omcZ*-deficient mutant was thin, with no pillar structures and an average thickness of 10 µm (Fig. 6). The level of protein present on the anode of the *omcZ* mutant was 0.09+/−0.002 mg/cm^2^, which is about 5 times higher than would be expected for wild type cells producing the equivalent amount of current , wild type cells exhibit a direct relationship between current production and both protein present on the anode surface and the thickness of the biofilm [20]. Transmission electron microscopy revealed that cells from the biofilm of the OmcZ-deficient mutant had pili (data not shown), indicating that the deletion of *omcZ* does not effect pili production. The biofilms of the complemented *omcZ* strain were similar to those of wild type, averaging 22 µm thick with a range of 8–37 µm when harvested at 10.7 mA, just at the end of log phase growth. As previously reported, the biofilm of the *pilA*-deficient mutant was thin, with no pillar structures and an average thickness of 5 µm [20]. The biofilms of the complemented *pilA* strain, when harvested at 14.2 mA, were an average of 40 µm thick with a range of 25–90 µm and were of similar thickness and structure as wild type biofilms (Fig. 6). Both the mutant deficient in *omcZ* and the mutant deficient in *pilA* formed biofilms similar to those of wild-type cells on the graphite surfaces when the electrodes were not connected to the cathode and fumarate was provided as the electron acceptor (Fig. 6).

## Discussion

These results demonstrate that *G. sulfurreducens* biofilms producing relatively high levels of current have substantial differences in their electron-transfer capabilities compared with biofilms growing on the same graphite surface, but with fumarate serving as the electron acceptor. These differences are associated with changes in expression of a small number of genes primarily encoding outer surface, electron-transfer proteins. Several of these genes, such as *pilA* [19], [20] and *omcB* [23], [41], have previously been shown to be important in extracellular electron transfer. However, one of the genes whose expression appears to be most up-regulated during growth on electrodes encodes OmcZ, an outer-surface *c*-type cytochrome that, as discussed in detail below, appears to play a unique role in high density current production as the only outer-surface c-type cytochrome essential for high density current production.

### Role of Pilin

The requirement for a functional PilA gene for high-density current production has been noted previously [20]. The finding presented here that expression of *pilA* is significantly higher in current-producing biofilms than during biofilm growth on the same surface, but without current production, is further circumstantial evidence for a role of pili in electron transfer through the biofilm.

The observation that the PilA-deficient mutant produced biofilms on glass or Fe(III) oxide-coated glass surfaces that were ca. half those of wild-type cells when fumarate was provided as an electron acceptor, led to the suggestion that the pili might also play an important structural role in biofilm formation [45]. However, in the studies reported here the PilA-deficient mutant produced biofilms as thick as those of wild-type cells on graphite surfaces. The disparity in these results could potentially be attributed to differences in the surfaces investigated and/or the fact that the previous studies investigated biofilms grown in static batch cultures whereas the cells growing on the graphite surface had a continual supply of new medium in a continuously stirred chamber. Regardless of the explanation, it is clear that the pilin are not required for thick biofilm formation on graphite. This result is consistent with, but does not prove, a conductive role for pili in the formation of thick biofilms on anode surfaces.

### Importance of OmcZ and other Outer-Surface Cytochromes in Current Production

The results suggest that the outer-membrane *c*-type cytochrome, OmcZ, is also an important component contributing to electron transfer through the thicker *G. sulfurreducens* biofilms required for high-density current production. The higher transcript abundance of *omcZ* in current-harvesting biofilms versus fumarate-grown biofilms was reflected in substantially more OmcZ in the current-harvesting biofilms. Most significantly, deletion of *omcZ* greatly decreased power production and *omcZ*-deficient cells did not adapt to overcome the loss of *omcZ*, even after relatively long incubations in current-harvesting mode. This suggests that *G. sulfurreducens* cannot readily adjust to the loss of OmcZ with the enhanced production of other outer-surface *c*-type cytochromes. OmcZ may function as the terminal cytochrome passing electrons to pilin, or may function as an electron carrier peripherally attached to the cell, in either case, OmcZ carries out a critical step, which can not be replaced by any of the other abundant cytochromes. Additional electrochemical studies have demonstrated that deletion of *omcZ* results in greater resistance to electron exchange between the cells and the anode, whereas deletion of the genes for other outer-surface *c*-type cytochromes, such as OmcS, OmcE, or OmcB does not have a similar effect [16]. This combination of results is consistent with OmcZ playing an integral part in electron transfer in *G. sulfurreducens* electrode biofilms. Other members of the *Geobacteraceae* do not have OmcZ homologs which may explain why these other species produce lower current densities than *G. sulfurreducens* [46, 47 & unpublished data].

This is the first study to suggest a role for OmcZ. In a series of previous studies, *omcZ* was not found to be differentially expressed under a variety of growth conditions [18], [41], [48]–[53]. Deletion of *omcZ* has no impact on Fe(III) oxide reduction. This finding, and the fact that the OmcZ-deficient mutant can produce some current in thin biofilms, suggests that OmcZ is not necessary for extracellular electron transfer when there is a close association between *G. sulfurreducens* and the extracellular electron acceptor. However, OmcZ is clearly important when long-range electron transfer through thick biofilms is required.

The greater abundance of *omcB* and *omcE* transcripts in current-producing biofilms suggest that OmcB and OmcE might be important in electron transfer to electrodes, but the studies in which the genes for these proteins were deleted demonstrated that they are not essential for current production. This is in accordance with previous results [18] at ca. 10-fold lower current densities. One possible explanation for these results is that the regulation of gene expression in *G. sulfurreducens* is unlikely to be optimized for electron transfer to graphite electrodes because it is unlikely that the *G. sulfurreducens* has previously faced selective pressure for optimal growth on electrodes [5]. Therefore, there may be changes in expression that are not necessarily required for improved current production. Alternatively, it was previously noted that an OmcB-deficient mutant could adapt to reduce Fe(III) via increased expression of genes for other *c*-type cytochromes [41]. Although initially deficient in Fe(III) oxide reduction, the *omcE*-deficient mutant adapts over time to reduce Fe(III) oxide [24]. An adaption period was also required for the *omcE*-deficient mutant to produce current as well as wild-type cells in low current density fuel cells [18]. Therefore, OmcB and OmcE might be components in electron transfer to electrodes in wild-type cells, but when their genes are deleted there may be other outer-surface proteins capable of electron transfer that provide alternative routes for electron transfer to electrodes.

Previous studies demonstrated that in the early colonization phase of electrodes (0.08–0.16 A/m^2^), there is a steady increase in transcripts of *omcS* [18], but the results shown here demonstrate that as current production and biofilm thickness increased transcript levels for *omcS* decreased. Although, deleting *omcS* inhibited current production in fuel cells in which electrochemical restrictions limited current production [18], as shown here, when these electrochemical limitations are eliminated, OmcS is not required for optimum current production.

In summary, in addition to providing further circumstantial evidence for the importance of microbial nanowires in high-density current production, this study provides several lines of evidence, which suggest that *G. sulfurreducens* requires OmcZ for maximum power production. One possibility is that OmcZ works in concert with the pili: OmcZ might function as an intermediary in electron flow to pili, which could serve as the ultimate conduit for long-range electron transfer; or OmcZ could be associated with the pili, contributing to their conductivity. Alternatively, the fact that OmcZ is displayed on the outer surface of the cell suggests that OmcZ in the extracellular matrix might make electron transfer among multiple OmcZ molecules a possible route for electron transfer through the biofilm. These possible mechanisms warrant further study.
